# Kaposi's Sarcoma-Associated Herpesvirus K7 Induces Viral G Protein-Coupled Receptor Degradation and Reduces Its Tumorigenicity

**DOI:** 10.1371/journal.ppat.1000157

**Published:** 2008-09-19

**Authors:** Hao Feng, Xiaonan Dong, Ashley Negaard, Pinghui Feng

**Affiliations:** Department of Microbiology, University of Texas Southwestern Medical Center, Dallas, Texas, United States of America; University of Southern California School of Medicine, United States of America

## Abstract

The Kaposi's sarcoma-associated herpesvirus (KSHV) genome encodes a G protein-coupled receptor (vGPCR). vGPCR is a ligand-independent, constitutively active signaling molecule that promotes cell growth and proliferation; however, it is not clear how vGPCR is negatively regulated. We report here that the KSHV K7 small membrane protein interacts with vGPCR and induces its degradation, thereby dampening vGPCR signaling. K7 interaction with vGPCR is readily detected in transiently transfected human cells. Mutational analyses reveal that the K7 transmembrane domain is necessary and sufficient for this interaction. Biochemical and confocal microscopy studies indicate that K7 retains vGPCR in the endoplasmic reticulum (ER) and induces vGPCR proteasomeal degradation. Indeed, the knockdown of K7 by shRNA-mediated silencing increases vGPCR protein expression in BCBL-1 cells that are induced for KSHV lytic replication. Interestingly, K7 expression significantly reduces vGPCR tumorigenicity in nude mice. These findings define a viral factor that negatively regulates vGPCR protein expression and reveal a post-translational event that modulates GPCR-dependent transformation and tumorigenicity.

## Author Summary

Kaposi's sarcoma-associated herpesvirus (KSHV) is the etiological agent of Kaposi's sarcoma. KSHV is also found in primary effusion lymphoma and multicentric Castleman's disease, rare lymphoproliferative diorders associated with immuno-suppression. The KSHV genome encodes a G protein-coupled receptor (vGPCR) that is believed to contribute to the KSHV-associated malignancies. vGPCR is a ligand-independent, constitutively active signaling molecule. It is not clear how vGPCR is negatively regulated. Here, we report that the KSHV small membrane K7 protein interacts with vGPCR through its putative transmembrane domain. Interaction with K7 retains vGPCR in the ER and facilitates its degradation by the proteasome, thereby reducing vGPCR protein expression. Consequently, K7 significantly reduces vGPCR-mediated transformation in vitro and tumor formation in nude mice. Our findings reveal that K7 functions as a viral factor to dampen vGPCR protein expression and negatively modulate the tumor-inducing capacity of vGPCR, implying that KSHV has evolved mechanisms to avoid deleterious effects and to permit persistent infection within its host.

## Introduction

Kaposi's sarcoma-associated herpesvirus (KSHV, also known as human herpesvirus 8) is believed to be the etiologic agent for Kaposi's sarcoma (KS) [1]. KSHV infection is also linked to primary effusion lymphoma [2] and multicentric Castleman's disease, rare lymphoproliferative malignancies of B-cell origin 3,4. The KSHV genome encodes over 80 viral polypeptides, many of which are capable of promoting cell proliferation and/or modulating host responses, when expressed in gene transfer experiments (for review see reference [5]). One such gene product consistently detected in KS lesions is the viral G protein-coupled receptor (vGPCR, or open reading frame 74) [6],[7].

vGPCR is a homolog of the human interleukin-8 receptor and possesses promiscuous chemokine-binding activity [8]. In tissue culture, vGPCR expression activates various signaling pathways and up-regulates the transcription of numerous cellular and viral genes that encode cytokines, signaling molecules, and transcription factors that culminate in promoting cell proliferation and endothelial tube formation [9]–[15]. Additionally, vGPCR transgenic mice developed tumors that resemble human KS lesions [9],[16],[17]. Although ligand binding is not required for vGPCR-mediated signaling, cognate chemokines appear to modulate vGPCR activity in tissue culture and in mice as well [8],[18]. Despite the fact that proliferative and prosurvival activities of vGPCR have attracted extensive attention in the past, accumulating evidence suggests that tightly regulated expression and signaling are important for vGPCR function in KSHV infection. Indeed, over-expression of vGPCR induced cell death in COS-1 cells and constitutive expression of vGPCR was toxic to PEL cells [6],[19]. Furthermore, vGPCR is predominantly translated from a bicistronic mRNA transcript downstream of K14 (vOX2), presumably reducing vGPCR protein expression [6],[20]. These observations suggest that KSHV likely has evolved mechanisms to achieve a temporary expression of the constitutively active vGPCR during lytic infection. A post-translational degradation is one of these mechanisms.

Regulated protein degradation is important for a variety of cellular events including cell cycle, apoptosis, signal transduction, immune response, and development. Cellular GPCR can be degraded either by the ubiquitin-proteasome system (UPS) or by the lysosome. Within the lysomsome, proteins are cleaved by diverse acidic proteases upon fusion with endosomes or autophagosomes. For UPS substrates, proteins destined for destruction are tagged with ubiquitin through sequential actions of the E1 activating enzyme, E2 conjugating enzyme, and E3 ligase [21]. Relying on the UPS, the endoplasmic reticulum (ER)-associated degradation (ERAD) pathway is a major route to remove mis-folded proteins post-translationally, and plays an essential role for ER quality control. Indeed, alteration of ERAD pathways has been implicated in diverse clinical presentations such as neurodegeneration and cystic fibrosis. Furthermore, viruses usurp components of this pathway to evade host recognition and possibly modulate other host responses [22]–[24].

We previously identified a small membrane protein, K7, which induces protein degradation of IκB and p53. K7 specifically interacts with the ubiquitin-associated domain of cellular protein linking integrin-associated protein and cytoskeleton (PLIC1) and antagonizes PLIC1, thereby promoting protein degradation [25]. Additionally, K7 was shown to deregulate cellular apoptosis by targeting Bcl-2 and an ER resident calcium modulating cyclophilin ligand [26],[27]. Although these data imply that K7 inhibits apoptosis to facilitate viral replication, its biological roles in KSHV infection remain obscure. We report here that K7 interacts with vGPCR and induces its proteasomeal degradation. The knockdown of K7 by shRNA-mediated silencing increased vGPCR protein expression in BCBL-1 cells that are induced for KSHV lytic replication. Biochemical and confocal microscopy analyses support that K7 retains vGPCR in the ER, thereby facilitating the proteasome to degrade vGPCR. Consequently, K7 significantly reduces vGPCR transformation in vitro and tumorigenicity in nude mice. These data establish a negative regulation of vGPCR protein expression and tumorigenicity by KSHV K7.

## Results

### K7 Interacts with KSHV vGPCR

**Figure 1:**
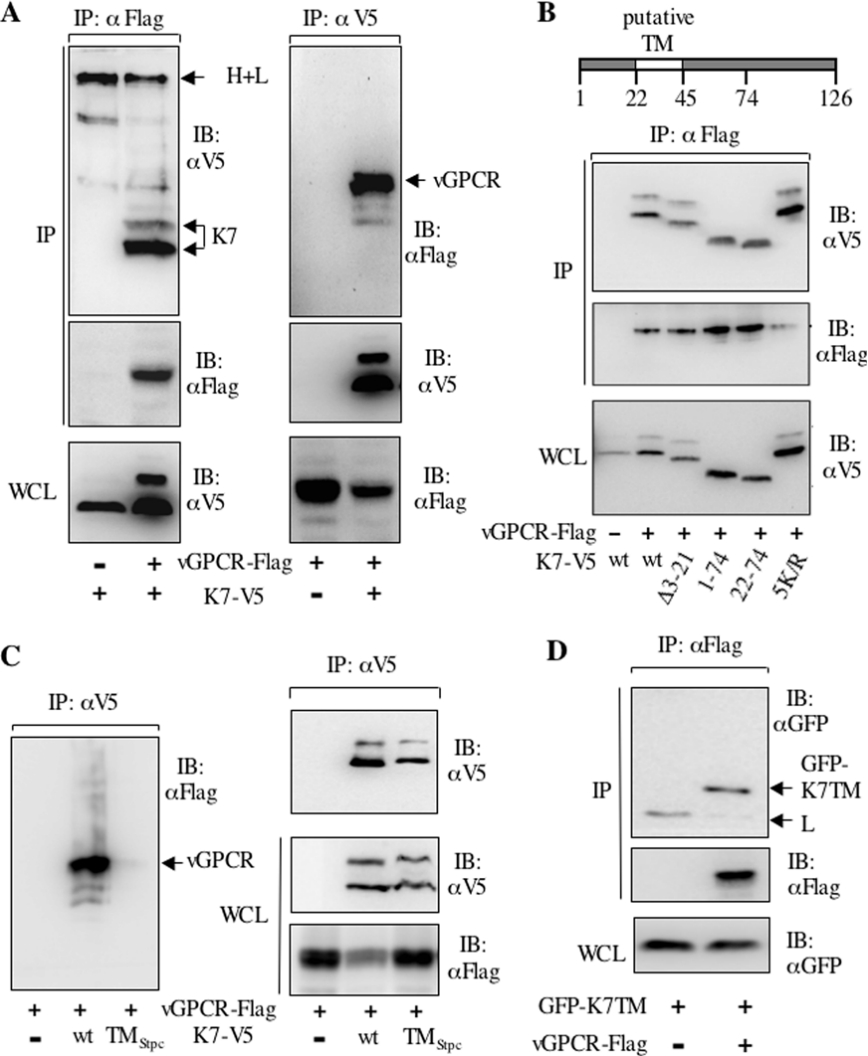
KSHV K7 interacts with vGPCR. (A) K7 interaction with vGPCR by co-IP. (Left) 293T cells were transfected with plasmids containing K7-V5 and vGPCR-Flag. Proteins precipitated with anti-Flag antibody were resolved by SDS-PAGE and analyzed by immunoblot with anti-V5 (top panel) and anti-Flag (middle panel) antibodies. WCLs were analyzed by immunoblot with anti-V5 (K7) antibody. Note: the K7 doublet indicates its glycosylated and unglycosylated forms. (Right) Proteins precipitated with anti-V5 antibody were analyzed by immunoblot with anti-Flag (top panel, peroxidase-conjugated) and anti-V5 (middle panel) antibodies. WCLs were analyzed by immunoblot with anti-Flag (vGPCR) antibody. H+L, the heavy and light chains of IgG; IB, immunoblot. (B) The K7 hydrophobic region is sufficient for its interaction with vGPCR. (Top) Diagram shows the structure of K7 protein and its residue numbers designed for the deletion analysis. Transfection of 293T cells with plasmids as indicated and IP with anti-Flag antibody were performed. Precipitated proteins were analyzed by immunoblot with anti-V5 (top panel) and anti-Flag (middle panel) antibodies. WCLs were analyzed with anti-V5 antibody. Δ3-21, deletion of amino acid 3 to 21; 5K/R, all lysine residues changed to arginine (for more details, please see [25]). (C) The putative K7 TM domain is necessary for its interaction with vGPCR. The putative K7 TM domain was replaced by the Stp C TM. Transfection of 293T cells with plasmids as indicated and IP were performed as in (A). Precipitated proteins were analyzed by immunoblot with anti-Flag (left panel) and anti-V5 (right top panel) antibodies. WCLs were analyzed by immunoblot with anti-V5 (right middle panel) anti-Flag (right bottom panel) antibodies. TM_StpC_ denotes the K7 mutant that contains a StpC TM domain. To achieve equivalent protein expression, 3-fold more plasmid containing K7TM_StpC_ than that containing the wt K7 was used for transfection. (D) The putative K7 TM domain is sufficient to interact with vGPCR. Transfection of 293T cells with plasmids as indicated and IP were performed as in (A). Precipitated proteins were analyzed by immunoblot with anti-GFP (top panel) and anti-Flag (middle panel) antibodies. WCLs were analyzed by immunoblot with anti-GFP antibody (bottom panel). L, the light chain of IgG.

To understand K7's functions, we searched for cellular interacting proteins with K7 as bait using the yeast two-hybrid screen. One clone contained a partial sequence of a putative G protein-coupled receptor that encodes its last four transmembrane (TM) domains. Since the KSHV genome encodes a vGPCR, we speculated that K7 interacts with vGPCR. To test this possibility, whole cell lysates of 293T cells transiently transfected with plasmids expressing vGPCR-Flag and/or K7-V5 were precipitated with the M2 anti-Flag antibody and precipitates were analyzed by immunoblot with anti-V5 (K7) antibody. Indeed, K7 was readily detected in immune complexes containing vGPCR (Figure 1A, left panels). Notably, vGPCR expression greatly increases K7 protein expression and the glycosylated form (the slower migration band) is only detected in the presence of vGPCR. Reciprocally, vGPCR was also precipitated by anti-V5 (K7) antibody (Figure 1A, right panels). Of note, the interaction between K7 and vGPCR was also identified by the yeast two-hybrid screen with a high throughput approach [28]. To further characterize the vGPCR-K7 interaction, K7 mutants that contain various deletions as described in our previous publications [25],[26] were used for a co-immunoprecipitation (co-IP) assay. The internal hydrophobic region (amino acid 22–74) containing the putative TM domain was sufficient to interact with vGPCR (Figure 1B). Unfortunately, K7 mutants lacking the TM domain were expressed at an undetectable level compared to the wild type (wt) K7. Thus, we failed to obtain any deletion mutant that no long interacts with vGPCR. Nevertheless, these data indicate that K7 interacts with vGPCR and suggest that its predicted TM domain is important for this interaction.

K7 contains a putative TM domain and vGPCR is a seven-membrane-spanning protein, therefore we examine whether the predicted K7 TM domain is necessary for this interaction. The K7 mutant whose putative TM region was replaced by a heterologous TM from the Saimiri transforming protein C (Stp C), designated K7TM_Stp C_, was constructed and expressed in 293T cells. We found that K7TM_Stp C_ failed to interact with vGPCR under the same co-IP conditions (Figure 1C). Of note, K7TM_Stp C_ was expressed and localized to intracellular organelles similarly to the wt K7 (unpublished data). Furthermore, appending the putative TM region (amino acids 23–45) of K7 to GFP renders it capable of binding vGPCR (Figure 1D). Thus, these data collectively support that K7 interacts with vGPCR and that the putative K7 TM region is necessary and sufficient for this interaction.

**Figure 2:**
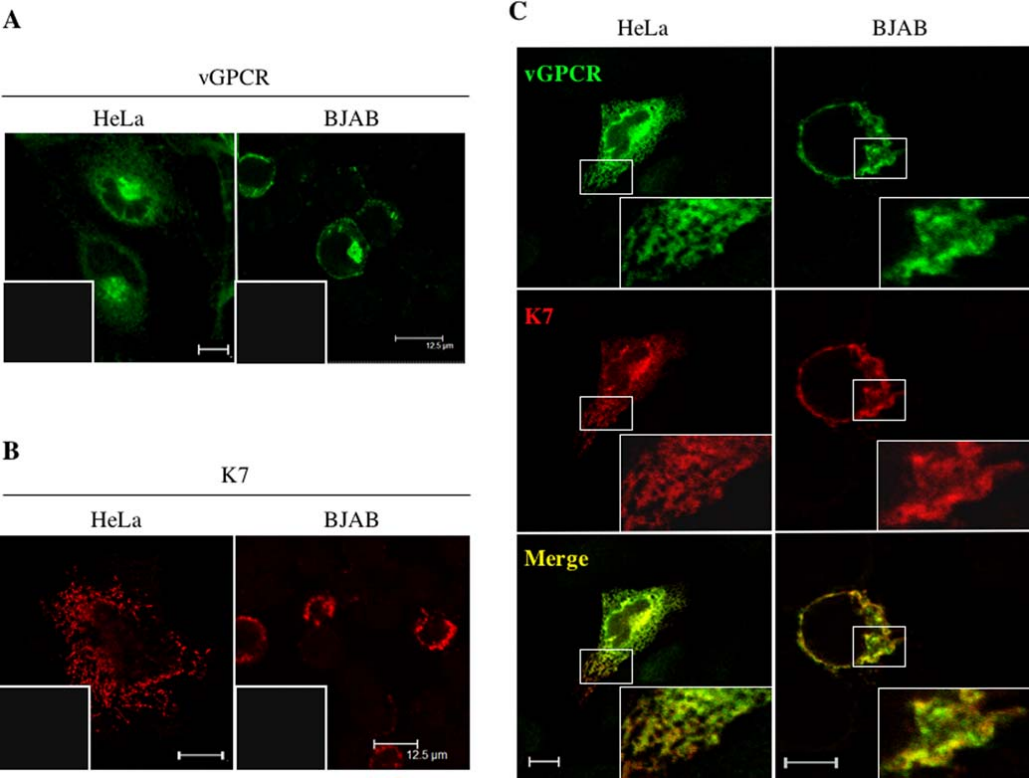
Intracellular localization of vGPCR and K7. Human HeLa and lymphoid BJAB cells were transfected as described in Materials and Methods. At 16 h after transfection, cells were fixed, permeabilized, and stained with rabbit anti-Flag and mouse anti-V5 antibodies. (A) vGPCR intracellular localization in human HeLa and BJAB cells. An inset in (A) and (B) represents an image of the other channel. (B) K7 intracellular localization in human HeLa and BJAB cells. (C) Intracellular co-localization of vGPCR (green) and K7 (red) in human HeLa and BJAB cells. Insets represent enlarged (3-fold) view of the boxed regions. Representative sections and their overlays (for panels in [C] only) are shown. Scale bar represents 12.5 µm.

K7 and vGPCR proteins are confined to distinct intracellular organelles. Particularly, vGPCR was reported to reside primarily in the trans-Golgi network (TGN) [7], whereas K7 localizes to both the ER and mitochondrial compartments [26],[27]. To examine the intracellular distribution of vGPCR and K7, indirect immuno-fluorescence microscopy was performed. To this end, human lymphoid BJAB and HeLa cells were transfected with plasmids expressing vGPCR-Flag and K7-V5, and analyzed by confocal microscopy. In both HeLa and BJAB cells, vGPCR predominantly localizes to a subcellular structure reminiscent of the TGN, while K7 distributes throughout the cytoplasm mainly as punctate vesicles (Figure 2A and 2B). In support of the interaction between K7 and vGPCR, K7 had an intracellular staining pattern similar to that of vGPCR in both HeLa and BJAB cells (Figure 2C). Despite the overall colocalization between K7 and vGPCR, there are some regions that either K7 or vGPCR is predominant, likely reflecting their distinct intracellular compartments that vGPCR and K7 reside in when they are separately expressed (Figure 2C, insets of BJAB cells).

### Overlapped Expression of vGPCR and K7

**Figure 3:**
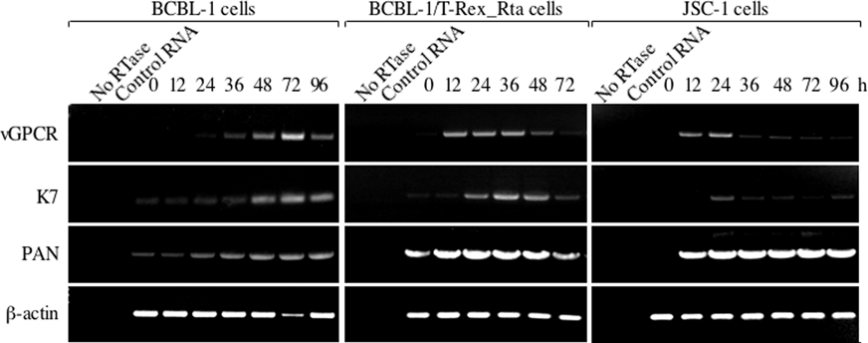
Overlapped expression of K7 and vGPCR during KSHV lytic replication. CBL-1, JSC-1, and BCBL-1/T-Rex_Rta cells were induced for KSHV lytic replication as described in Materials and Methods. RT-PCR analyses were performed using primers for vGPCR, K7, PAN, and cellular β-actin. No reverse transcriptase reaction was performed with total RNA of 36 h after treatment of each panel; h, hour after treatment.

The fact that K7 interacts with vGPCR prompted us to investigate the temporal expression kinetics of K7 and vGPCR in KSHV lytic replication. Both K7 and vGPCR were reported to be expressed early during KSHV lytic reactivation and/or de novo infection [6],[7],[27],[29]; however, the relative temporal expression of vGPCR and K7 remains unclear. Our interaction study suggests that vGPCR and K7 are possibly expressed at the same time. Thus, we examined mRNA levels of vGPCR and K7 by reverse-transcriptase (RT)-polymerase chain reaction (PCR). The KSHV latently infected PEL cell lines BCBL-1 (KSHV only) and JSC-1 (KSHV and EBV co-infected) were treated with TPA to induce KSHV lytic replication. Alternatively, lytic replication was reactivated by Rta expression that was induced by doxycycline using the BCBL-1/T-Rex_Rta cell line [30]. RT-PCR analyses were performed using primers specific for vGPCR, K7, the polyadenylated nuclear RNA (PAN), and cellular β-actin. When treated with TPA (20 ng/ml), lytic replication was initiated in both BCBL-1 and JSC-1 cells which was indicated by potent induction of PAN transcripts (Figure 3). The residual PAN RNA in untreated BCBL-1 cells and BCBL-1/T-Rex_Rta cells (lane 3 of left two sets in Figure 3) are likely due to spontaneous lytic replication of KSHV or leaky Rta expression in these PEL cells, respectively. Upon TPA induction, vGPCR transcripts peaked at 72 h, which coincided with the highest mRNA level of K7 in BCBL-1 cells. Upon Rta expression induced by doxycycline addition, vGPCR was highly expressed as early as 12 h post induction and was sustained for more than 24 h (Figure 3, middle panels), while K7 transcripts gradually increased and peaked at 36 h after TPA induction when vGPCR mRNA started to decline. This indicates that K7 expression predominantly overlaps with that of vGPCR in response to the KSHV lytic switch protein, Rta. Similar results were obtained for TPA-induced JSC-1 cells in which vGPCR was highly expressed at 12 and 24 h after treatment. Meanwhile, K7 was highly expressed at 24 h after induction (Figure 3, right panels). The most abundant lytic transcript of KSHV, PAN, was significantly induced by TPA and sustained high transcript levels in BCBL-1 and JSC-1 cells throughout the entire induction period. This was more pronounced by Rta induction (Figure 3 third panel from top), while cellular β-actin transcript remained the same. Overall, these data indicated that K7 and vGPCR are expressed at the same time and suggest that the interaction between these two molecules is biologically relevant.

### K7 Reduces vGPCR Protein Expression

**Figure 4:**
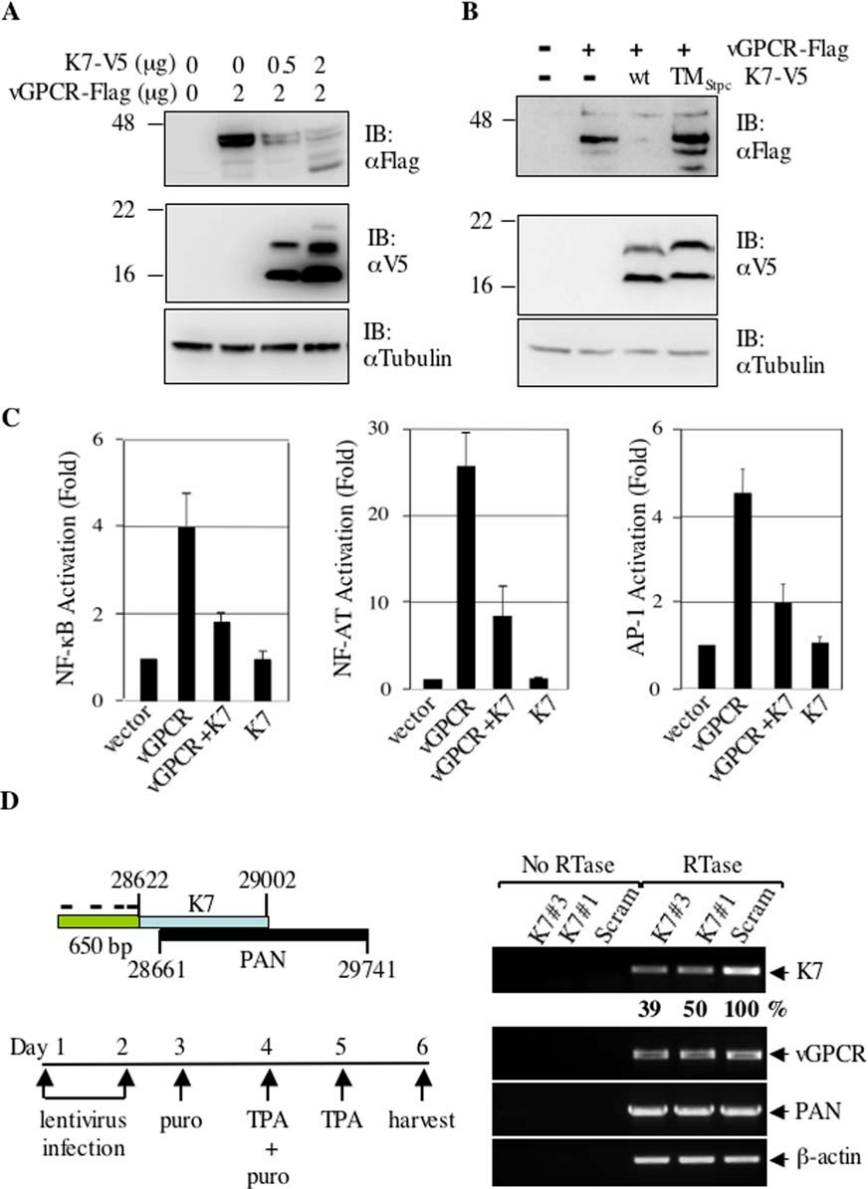
K7 reduces vGPCR protein expression. (A) K7 reduces vGPCR protein expression in a dose-dependent manner. Human endothelial ECV cells were transfected with plasmids containing vGPCR-Flag and K7-V5 as indicated. WCLs were analyzed by immunoblot with anti-Flag (vGPCR, top panel), anti-V5 (K7, middle panel), and anti-tubulin (bottom panel) antibodies. (B) The putative K7 TM domain is necessary for vGPCR downregulation. Transfection of ECV cells and immunoblot analyses were performed as in (A). Data represents three independent experiments. (C) K7 reduces vGPCR-mediated activation of NF-κB, NF-AT, and AP-1 transcription factors. 293T cells were transfected with reporter plasmid cocktail, and plasmids containing vGPCR and K7. Luciferase activity normalized against β-galactosidase activity is shown. Error bars denote standard deviation and data represent three independent experiments. (D) K7 knockdown by shRNA-mediated silencing. (Left Top) The relative genomic locations of K7 coding sequence and the transcribed region of PAN were shown. The numbers indicate the nucleotide position according to a published KSHV genome sequence (accession number: U75698). Bars represent relative location of sequences base paired with four shRNAs within the 5′ untranslated region of K7. The order of shRNAs on the diagram is: shRNA#2, #3, #1, and #4. (Left Bottom) A diagram shows the experimental design of lentivirus infection and KSHV lytic replication induced by TPA. (Right) RT-PCR analyses were performed using gene specific primers for K7, β-actin, vGPCR, and PAN as described in Materials and Methods. Numbers indicate intensity of K7 band measured by densitometry. Data represent two independent experiments.

We have consistently observed that K7 co-expression significantly reduces the protein level of vGPCR (Figure 1A and 1B), suggesting that K7 modulates vGPCR biosynthesis. Because our previous data implicate K7 in regulating protein degradation [25], we speculated that K7 induces the degradation of vGPCR. To examine K7's effect on vGPCR protein expression, human endothelial ECV cells were transiently transfected with a plasmid expressing vGPCR-Flag and increasing amounts of a plasmid expressing K7-V5. Whole cell lysates were analyzed by immunoblot for vGPCR protein expression. The result shows that K7 reduces vGPCR protein in a dose-dependent manner (Figure 4A). The specificity of K7 is further supported by the observation that the K7TM_Stp C_ chimera, a mutation that abolished its interaction with vGPCR, failed to suppress vGPCR protein expression (Figure 4B).

Previous publications have convincingly shown that vGPCR activates a number of signaling pathways, leading to the activation of NF-AT, NF-κB, and AP-1 transcription factors [19],[31],[32]. To further correlate K7's effect on vGPCR protein expression, the transcription activation of NF-AT, NF-κB, and AP-1 response elements by vGPCR were measured by luciferase assays in transiently transfected 293T cells. Consistent with published data, vGPCR activated NF-κB, NF-AT, and AP-1 transcription factors by approximately 4, 25, and 4.5 fold, respectively. In contrast, K7 exhibited no effect on the transcription of NF-κB, NF-AT, and AP-1 (Figure 4C). In agreement with our observation that K7 reduces vGPCR protein, K7 suppressed the transcription activation by vGPCR to approximately two-fold for NF-κB and AP-1, and eight-fold for NF-AT, respectively (Figure 4C). These data indicate that K7 reduces vGPCR protein expression and mitigates vGPCR-activated downstream signaling.

**Figure 5:**
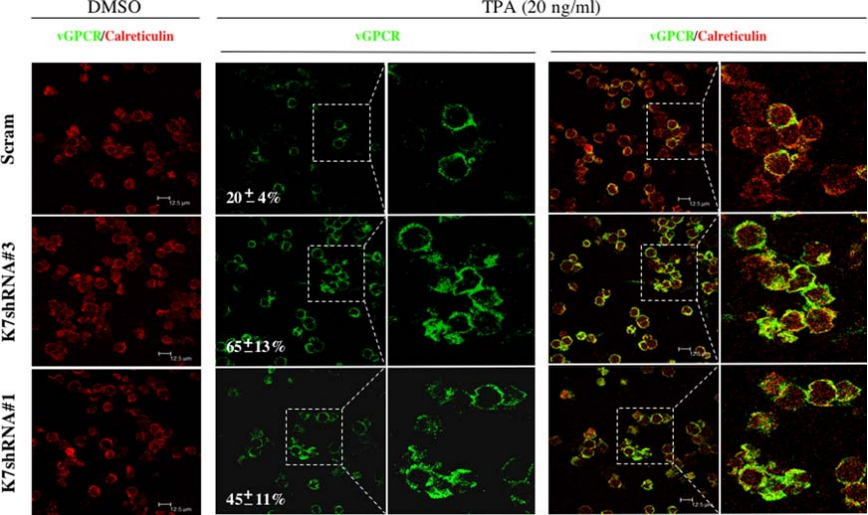
Increased vGPCR protein expression by shRNA-mediated K7 knockdown. Lentivirus infection and KSHV lytic replication induced with TPA were performed as in Figure 4D. Cells were fixed and stained with anti-vGPCR (green) and anti-calreticulin (red) antibodies. vGPCR-positive and vGPCR-negative cells in 5 randomly selected fields were counted to obtain the percentage shown in the middle panels. For BCBL-1 cells induced with TPA, images to their right represent enlarged (2.5-fold) view of the boxed regions. Representative sections and their overlays are shown. Scale bar represents 12.5 µm.

Although our studies clearly indicate that K7 reduces vGPCR protein expression, these experiments relied on exogenous protein expression. To corroborate K7-reduced vGPCR protein expression during KSHV infection, the shRNA-mediated silencing experiments were designed to knock down K7 expression and vGPCR protein level was examined by confocal microscopy. Both K7 and vGPCR are expressed in the lytic phase during KSHV infection. Given the fact that K7 open reading frame overlaps with the transcribed region of PAN (or T1.1), four pairs of short hairpin RNA (shRNA) molecules targeting the 5′ untranslated region of K7 transcripts were cloned (Figure 4D) and lentiviral particles were produced in 293T cells. Lentivirus was then used to infect KSHV-positive BCBL-1 cells that were subsequently treated with TPA to induce KSHV lytic replication. A scrambled shRNA was used as a control for all silencing experiments. Among the shRNAs, K7 shRNA#1 and #3 significantly reduced the level of K7 transcripts, while these two shRNA molecules had no discernable alteration on mRNA levels of PAN and vGPCR, when compared to BCBL-1 cells expressing the scrambled shRNA (Figure 4D, right panels). Densitometry of RT-PCR products showed that K7 shRNA#3 and shRNA#1 had a silencing efficiency of 60% and 50% (Figure 4D). Semi-quantitative PCR analyses using serial dilution of cDNA templates further support that K7 transcripts were reduced by 60%–70% (Figure S1). Notably, the knockdown of K7 did not significantly affect cell viability after lytic induction, suggesting that additional viral proteins such as vBcl-2 and vFLIP play a redundant antiapoptotic role. BCBL-1 cells infected with lentiviruses expressing K7 shRNA#1, shRNA#3, or the scrambled shRNA were induced with TPA for KSHV lytic replication. At 48 h after induction, cells were fixed and subjected to confocal microscopy analysis to examine vGPCR protein level. As shown in Figure 5, the knockdown of K7 significantly increased vGPCR protein expression (second and third rows from the top), while the ER resident protein calreticulin was not affected. The vGPCR-positive cells increased from 20% in BCBL-1 cells expressing the scrambled shRNA to 65% in BCBL-1 cells expressing K7 shRNA#3 and 45% in BCBL-1 cells expressing K7 shRNA#1 (Figure 5, middle panels). Furthermore, merged images clearly indicate the increased vGPCR protein expression upon K7 knockdown, because image color shifted from red (calreticulin) in BCBL-1 cells expressing the scrambled shRNA to green (vGPCR) in BCBL-1 cells expressing K7 shRNA (Figure 5, right panels). Taken together, these findings support the conclusion that K7 suppresses vGPCR protein expression in tissue culture and in KSHV lytic infection.

### K7 Induces Proteasome-Dependent Degradation of vGPCR

**Figure 6:**
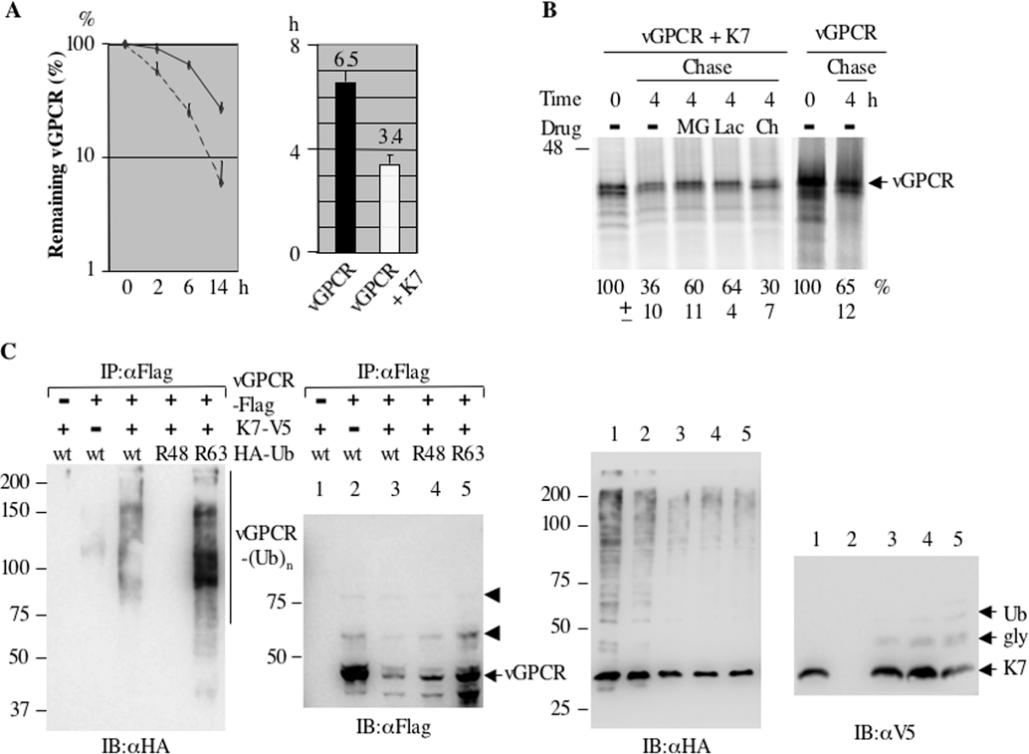
K7 induces proteasome-dependent degradation of vGPCR. (A) K7 reduces the half-life of vGPCR. ECV cells were transfected with plasmids expressing vGPCR-Flag and K7-V5. Pulse chase, IP, and autoradiography analyses were performed as described in Materials and Methods. The fully glycosylated vGPCR band was quantified and its half-life was calculated. Data (left panel) represent three independent experiments and error bars denote standard deviation. (B) K7-induced vGPCR degradation is dependent on the proteasome. Transfection and pulse chase experiments with ECV cells were performed as described in (A) except cells were harvested at time points as indicated. The numbers at the bottom indicate the relative intensity (top row) of vGPCR band compared to the initial chase time point and standard deviation (bottom row). Data represent three independent experiments. Lac: lactacystin (10 µM); MG: MG132 (20 µM); Ch: chloroquine (50 µM). (C) K7 increases vGPCR ubiquitination. NIH3T3/lenti-puro (Vec) or NIH3T3/lent-vGPCR-Flag (vGPCR) were transfected with plasmids expressing K7-V5 and HA-tagged Ubiquitin (wt), K48R (R48), or K63R (R63). At 36 h after transfection, cells were treated with lactacystin (20 µM) for 6 h. vGPCR was precipitated with anti-Flag sepharose and eluted with Flag peptide for immunoblot with anti-HA (ubiquitin, first panel from left), or eluted with loading buffer for immunoblot with anti-Flag (vGPCR, second panel). WCLs were analyzed by immunoblot with anti-HA (ubiquitin; third panel) and anti-V5 (K7; fourth panel) antibodies. Arrowheads indicated ubiquitinated vGPCR species (second panel).

Our previous publication indicated that K7 induces protein degradation dependent on the UPS [25]. To investigate the mechanism by which K7 downregulates vGPCR protein expression, the half-life of vGPCR was measured by a pulse chase experiment. Transient transfection of ECV cells expressing vGPCR or vGPCR and K7 were pulse labeled with [^35^S]-methionine/cysteine (Met/Cys). After extensive washing, ECV cells were chased with cold medium. Precipitated vGPCR was quantified by autoradiography and its half-life was calculated. As shown in Figure 6A, vGPCR has a half-life of about 6.5 h and K7 expression reduced its half-life to approximately 3.4 h, indicating that K7 promotes vGPCR degradation. Cellular GPCRs are 7-membrane-spanning proteins that can be degraded through the lysosome or the UPS [33]. To examine whether K7-induced vGPCR degradation is dependent on the proteasome or the lysosome, vGPCR protein stability was examined by a pulse chase experiment with either a lysosome inhibitor (chloroquine) or proteosome inhibitors (lactacystin and MG132). It was found that lactacystin and MG132, but not chloroquine, completely blocked K7-induced vGPCR degradation, indicating that this process relies on the proteolytic activity of the proteasome (Figure 6B).

Proteasome substrates are often marked with polyubiquitin chains that facilitate delivery to and subsequent degradation by the proteasome. To further corroborate the proteasome-dependence of K7-induced vGPCR degradation, vGPCR ubiquitination was examined by immunoprecipitation and immunoblot. vGPCR was precipitated with anti-Flag sepharose and analyzed by immunoblot with anti-HA (ubiquitin) antibody. Consistent with the increased degradation of vGPCR, K7 promoted vGPCR polyubiquitination in the presence of a proteasome inhibitor, lactacystin (Figure 6C, first panel from left). Recent findings have shown that K48-linkage ubiquitin chains mediate protein degradation and K63-linkage ubiquitin chains are involved in signal transduction. Thus, these ubiquitin mutants were included in the vGPCR ubiquitination assay. Indeed, the K48R mutant, but not the K63R mutant, completely abolished vGPCR ubiquitination induced by K7 (Figure 6C). Of note, the protein level of precipitated vGPCR and vGPCR in whole cell lysate in the presence of K7 is significantly lower than vGPCR alone (Figure 6C, second panel, lanes 2–5, and Figure S2). These data collectively support the conclusion that K7 increases vGPCR ubiquitination and promotes its proteasomeal degradation.

### K7 Retains vGPCR in the ER to Induce its Degradation

**Figure 7:**
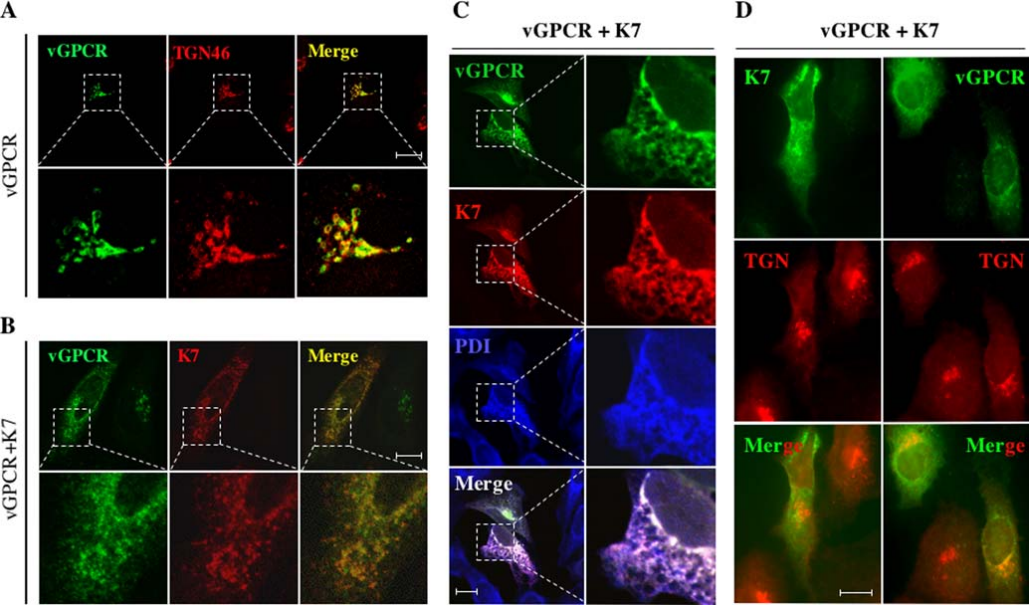
K7 retains vGPCR in the ER. (A) vGPCR localizes to the TGN. HeLa cells were transfected with plasmids containing vGPCR-Flag. At 16 h after transfection, cells were fixed and stained with mouse anti-Flag (vGPCR, green) and sheep anti-TGN46 (red) antibodies. For both (A) and (B), images at the bottom represent enlarged (3-fold) view of the boxed regions. Representative sections and their overlays are shown. Scale bar represents 12.5 µm. (B) K7 alters vGPCR intracellular localization. HeLa cells were transfected with plasmids containing vGPCR-Flag and K7-V5, and fixed as in (A). Cells were stained with rabbit anti-Flag (vGPCR, green) and mouse anti-V5 (K7, red) antibodies. (C) K7 retains vGPCR in the ER. HeLa cells were transfected with plasmids expressing HA-vGPCR and K7-V5, and fixed as described in (A). Cells were stained with rabbit antibody to protein disulfide isomerase (PDI, blue) and mouse anti-V5 (K7, red) antibody. After staining with corresponding secondary antibody and extensive washing, cells were further stained with Alexa 488-conjugated anti-HA antibody (vGPCR, green). Images on the right represent enlarged (3-fold) view of the boxed regions. Representative sections and their overlays are shown. Scale bar represents 12.5 µm. (D) The intracellular localization of K7 and vGPCR in relation to the TGN. HeLa cells were transfected with plasmids containing vGPCR-Flag and K7-V5. Cells were fixed and stained with mouse monoclonal anti-V5 (K7, green) and sheep anti-TGN46 (red) (left panels), or rabbit polyclonal anti-Flag (vGPCR, green) and sheep anti-TGN (red) (right panels). Representative images and their overlays are shown. Scale bar represents 12.5 µm.

To further define the molecular action of K7 in inducing vGPCR degradation, vGPCR intracellular localization was analyzed by confocal microscopy using human HeLa cells. Consistent with a previous report [7], vGPCR primarily localized to the TGN stained by anti-TGN46 antibody (Figure 7A). Upon K7 expression, vGPCR localized to intracellular structures that resemble the ER and nuclear membrane (Figure 7B), suggesting that K7 retains vGPCR in the ER compartment. Indeed, HeLa cells expressing both K7 and vGPCR revealed that these two proteins colocalized significantly with protein disulfide isomerase (PDI), an ER resident protein (Figure 7C), supporting the notion that K7 retains vGPCR in the ER. Furthermore, K7 expression reduced vGPCR localization in the TGN when intracellular distribution of vGPCR and K7 was examined in relation to TGN46 (Figure 7D). These results clearly indicate that K7 retains vGPCR in the ER and suggest that K7 induces vGPCR degradation via the ER-associated degradation pathway.

### vGPCR and K7 Inhibit Cell Growth in vitro

**Figure 8:**
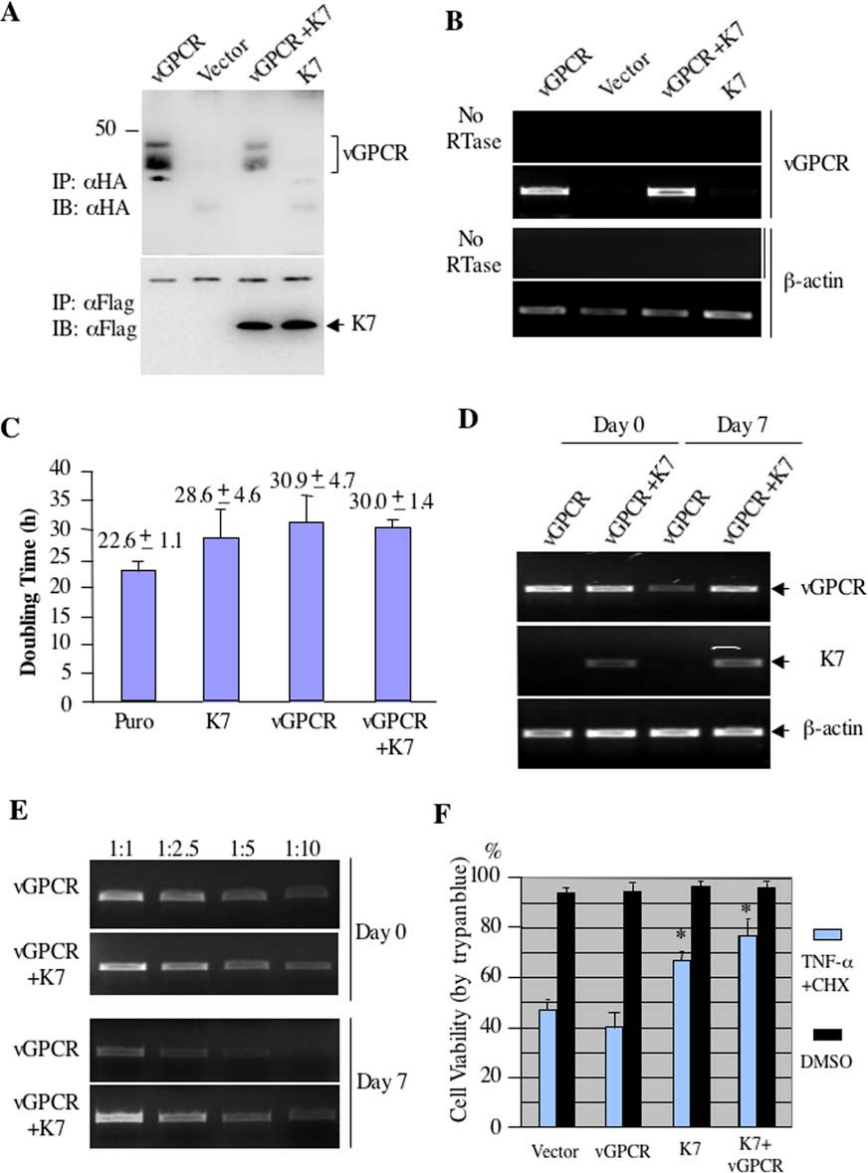
The effect of vGPCR and K7 on cell growth in vitro. (A) K7 reduces vGPCR protein expression in stable NIH3T3 cells. Whole cell lysates of NIH3T3/vector, NIH3T3/vGPCR, NIH3T3/K7, and NIH3T3/vGPCR+K7 were precipitated with anti-HA and immunoblotted with anti-HA antibody (top panel). For K7 expression, above stable cells were treated with MG132 for 6 h before harvest, K7 was precipitated with anti-Flag and analyzed by immunoblot with anti-Flag antibody (bottom panel). (B) K7 does not reduce vGPCR mRNA level. The mRNA level of vGPCR in stable cell lines as described in (A) was analyzed by RT-PCR and β-actin PCR product serves as a loading control. (C) vGPCR and K7 inhibit cell growth. NIH3T3 stable cell lines described in (A) were cultured in complete DMEM containing puromycin (1 µg/ml) and counted at 24 h and 48 h. The doubling time was measured as derscribed in Materials and Methods. Data represent three independent measurements and error bars denote standard deviation. (D) K7 reduces the loss of vGPCR transcripts in NIH3T3 cells. NIH3T3 stable cell lines as described in (A) were passaged up to 7 d and RT-PCR analyses were performed with primers specific for vGPCR, β-actin, and K7. (E) PCR analyses with serial dilution of cDNA templates from NIH3T3/vGPCR and NIH3T3/vGPCR+K7 were performed using vGPCR-specific primers. The ratio denotes fold of serial dilutions. (F) The effect of vGPCR and K7 on apoptosis. NIH3T3 stable cell lines were treated with TNF-α (5 ng/ml) and CHX (1 µg/ml) for 24 h; cell viability measured by trypan blue staining is shown. Data represent 3 independent experiments, and error bars denote standard deviation; *p<0.03 relative to NIH3T3/vector cells as calculated by Student's *t*-test.

To examine K7's effect on vGPCR biological functions, NIH3T3 cell lines stably expressing K7, vGPCR, and vGPCR+K7 were established with lentivirus infection. As shown in Figure 8A, K7 detectably reduced vGPCR protein expression without affecting its mRNA levels (Figure 8B). Of note, vGPCR did not further increase K7 protein after treatment by the proteasome inhibitor MG132 (Figure 8A). During the course to establish these stable cell lines, we noticed that NIH3T3 cells expressing vGPCR grow more slowly than the control NIH3T3 cells. In contrast to what was reported [34], NIH3T3/vGPCR cells had a doubling time of approximately 31 h that is significantly longer than 22.6 h of NIH3T3/vector cells. RT-PCR analysis indicated that vGPCR is expressed at similar levels in NIH3T3, and reactivated BCBL-1 and JSC-1 cells (Figure S3). This observation rules out the possibility that the inhibitory effect on cell growth is due to over-expression. Interestingly, K7 expression also increased NIH3T3 doubling time to roughly 28.2 h. Consistent with K7-reduced vGPCR protein expression, K7 co-expression slightly decreases the doubling time of NIH3T3 cells to 30 h (Figure 8C). Due to K7's inhibitory effect on cell growth and vGPCR-increased K7 expression (unpublished data), the subtle difference in cell growth may be significant. Given the inhibitory effect of vGPCR on cell growth, we suspect that NIH3T3 cells expressing higher vGPCR will gradually decrease when continuously cultured without selection. To test this, NIH3T3/vGPCR and NIH3T3/vGPCR+K7 cells were passaged for a week and RT-PCR analyses were performed to assess the mRNA levels of vGPCR. Indeed, the vGPCR mRNA level significantly decreased after 1 wk of passage and K7 reduced the vGPCR loss (Figure 8D). Semi-quantitative RT-PCR and real-time PCR analyses revealed that the vGPCR mRNA in NIH3T3/vGPCR+K7 was approximately 5-fold of that in NIH3T3/vGPCR cells at day 7 (Figure 8E and S4). The rapid loss of vGPCR transcripts suggests that NIH3T3 cells that lost vGPCR have a growth advantage.

We and others have shown that K7 inhibits apoptosis induced by various stress stimulations [25]–[27]. To examine whether vGPCR affects K7's antiapoptotic function, NIH3T3 stable cells were stimulated with TNF-α and cyclohexamide and cell viability was measured by trypan blue staining as described previously [25]. It was found that vGPCR expression had no significant effect on cell survival upon TNF-α stimulation, while K7 expression increased cell survival rate by 20% compared to NIH3T3/vector cells (Figure 8F). Interestingly, vGPCR co-expression with K7 further promotes cell survival rate by approximately 30%, indicating that vGPCR potentiates K7's antiapoptotic effect. This is consistent with our observation that vGPCR increases K7 protein expression (unpublished data). These results indicate that K7 reduces vGPCR-induced stress and suggest that K7 likely co-operates with vGPCR to promote cell survival during KSHV lytic replication.

### K7 Negatively Regulates vGPCR Tumorigenicity

**Figure 9:**
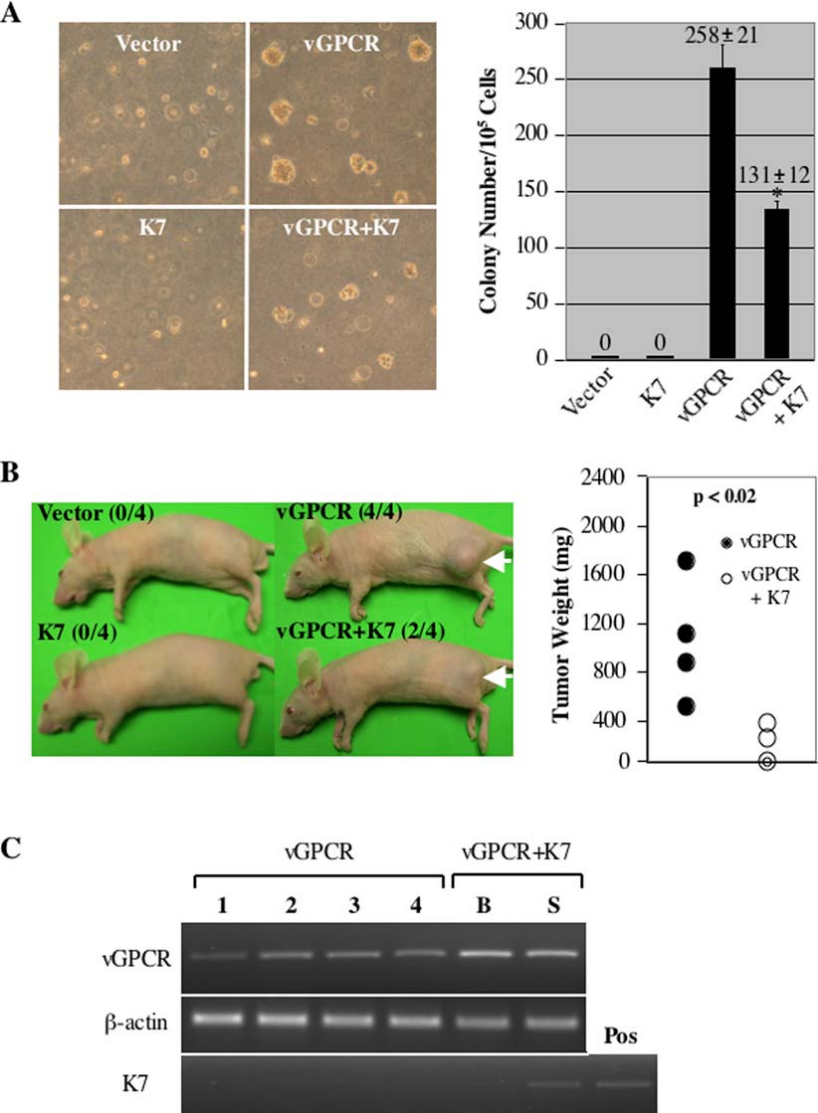
K7 negatively regulates vGPCR tumorigenicity. (A) K7 reduces vGPCR activity to stimulate anchorage-independent growth of NIH3T3 cells. Colonies under microscope were photographed (left panels, 4×) or counted (right graph) after a 2-wk incubation. Data represent 3 independent experiments. Error bars denote standard deviation; *p<0.02 relative to NIH3T3/vGPCR cells as calculated by Student's *t*-test. (B) K7 reduces vGPCR tumorigenicity in nude mice. Cells were injected into nude mice subcutaneously, and mice were killed and photographed (left panel) 6 wk later. Tumor weight was measured (right graph). The numbers in parenthesis indicate the number of mice developed tumor among 4 tested animals. Arrows indicate location of tumor and data represent 4 independent measurements for each group. (C) vGPCR and K7 expression in tumors. RT-PCR analyses were performed as described in Materials and Methods using primers specific for vGPCR, K7, and cellular β-actin. PCR products resolved on agarose gel were photographed. B, the bigger tumor; S, the smaller tumor; Pos, a positive control of K7.

In a mouse pathogenesis model, vGPCR is sufficient to induce tumor formation in nude mice and vGPCR transgenic mice developed lesions that resemble human KS, suggesting its potential contribution to KSHV-associated malignancies [17],[18],[34]. To assess K7's effect on vGPCR tumorigenicity, NIH3T3 stable cells expressing K7, vGPCR, or vGPCR+K7 were mixed with NIH3T3 cells and colony formation on soft agar was examined. Similar to the human cytomegalovirus US28 [35], vGPCR-expressing cells stimulated anchorage-independent growth of NIH3T3 cells, whereas neither NIH3T3/vector, nor NIH3T3/K7 cells supported colony formation (Figure 9A). In support of the observation that K7 suppressed vGPCR protein expression, NIH3T3/vGPCR+K7 cells formed smaller colonies than NIH3T3/vGPCR cells (Figure 9A, left panels). Furthermore, K7 expression also reduced the number of colonies from 258 of NIH3T3/vGPCR to 131 of NIH3T3/vGPCR+K7 (Figure 9A, right diagram). To further investigate K7's effect on vGPCR tumorigenicity in vivo, these stably transfected cells were injected into nude mice and tumor growth was assessed. Mice injected with NIH3T3/vGPCR developed visible tumors within two weeks and all mice harbored tumors after 6 wk. Neither NIH3T3/vector cells nor NIH3T3/K7 cells induced apparent tumor in nude mice. In agreement with results from the soft agar assay, K7 significantly reduced vGPCR capacity to promote tumor growth in nude mice as shown by the number of mice harboring tumor and tumor weight (Figure 9B). All four nude mice injected with NIH3T3/vGPCR developed tumors after 6 wk, whereas only two mice injected with NIH3T3/vGPCR+K7 developed tumors, which were substantially smaller (Figure 9B). The mean weight of tumors derived from NIH3T3/vGPCR cells is approximately 8-fold higher than that of tumors derived from NIH3T3/vGPCR+K7 cells (Figure 9B and unpublished data). Interestingly, we found that K7 transcripts were expressed at a higher level in the smaller tumor than the bigger tumor, suggesting that K7 inhibits the vGPCR-dependent tumor growth in vivo (Figure 9C). This result is consistent with the observation that K7 expression reduces vGPCR tumorigenicity (Figure 9B). In contrast, the vGPCR transcript was expressed more abundantly in tumors derived from NIH3T3/vGPCR+K7 cells than those derived from NIH3T3/vGPCR cells (Figure 9C). This likely represents the relative expression of vGPCR in stable NIH3T3 cells before mice injection. Overall, K7 negatively regulates vGPCR tumorigenicity in vitro by a soft agar assay and in vivo in nude mice.

## Discussion

We report here that KSHV K7 interacts specifically with vGPCR and induces the rapid degradation of vGPCR, thereby reducing vGPCR protein expression. The putative K7 TM domain is necessary and sufficient for its interaction with vGPCR, indicating a specific interaction between vGPCR and K7. However, the K7/vGPCR interaction may involve multiple residues within the putative TM domain of K7 because further mutational analyses within this domain failed to identify critical residues that are essential for this interaction (unpublished data). Alternatively, additional cellular components such as membrane proteins or lipids could be involved, as our co-IP procedure does not exclude this possibility. Nevertheless, these data support the conclusion that K7 interacts specifically with vGPCR.

We have previously shown that K7 antagonizes cellular PLIC1, a factor that inhibits proteasome-mediated protein degradation, and induces rapid degradation of p53 and IκB [25]. Our current study enlists vGPCR as an additional proteasome substrate whose degradation is accelerated by K7. The specificity of K7-induced degradation appears to be derived from an interaction with either a proteasome substrate such as vGPCR or a key component of the UPS pathway such as PLIC1. It is possible that binding of K7 to cellular PLIC1 also contributes to K7-dependent reduced expression of vGPCR, given that PLIC1 has been shown to promote protein expression of multiple transmembrane proteins [36],[37]. Indeed, we have observed that PLIC1 overexpression increases vGPCR protein, while the knockdown of PLIC1 by shRNA-mediated silencing greatly reduces vGPCR protein expression. These data indicate that PLIC1 is a positive regulator for vGPCR expression (unpublished data). Future experiments will determine whether K7 binding to PLIC1 is sufficient for suppressing vGPCR protein expression.

Confocal microscopy analyses and biochemical assays examining vGPCR protein degradation support the conclusion that K7 retains vGPCR in the ER and allows vGPCR to be removed by the proteasome. The rapid degradation of vGPCR induced by K7 also correlates with increased ubiquitination upon treatment with a proteasome inhibitor. vGPCR appears to carry polyubiquitin chains and K7-induced polyubiquitination of vGPCR is specifically inhibited by the K48R ubiquitin mutant, but not by the K63R ubiquitin mutant (Figure 6C). Interestingly, the K63R mutant significantly increased unmodified- as well as ubiquitinated-vGPCR protein. This is likely due to the inhibitory effect of K63R ubiquitin on vGPCR signaling that is presumably coupled to vGPCR degradation. For example, the K63R mutant may inhibit signaling downstream vGPCR such as NF-κB activation, therefore stabilizing vGPCR. Alternatively, vGPCR polyubiquitin chains may contain a mixture of K63- and K48-linkages. The fact that the K48R mutant abolished, while the K63R mutant increased vGPCR ubiquitination suggests that K48-linkage is necessary to initiate ubiquitination, whereas K63-linkage is important for degradation. These intriguing possibilities are not mutually exclusive and require further experimental investigation. Our data, however, do not exclude the possibility that vGPCR undergoes ubquitination-independent proteasomeal degradation. In transfected cells, K7 consistently altered vGPCR intracellular distribution, showing a more diffused ER/nuclear membrane pattern that was confirmed by staining with anti-PDI antibody. This observation suggests that K7 retains vGPCR in the ER in order to induce vGPCR degradation. This also implies that K7 likely engages the ERAD pathway to facilitate vGPCR degradation in similar ways employed by human cytomegalovirus US11 and murine γ-herpesvirus 68 mK3 [23],[24],[38],[39]. Future experiments will be directed to test whether K7-induced protein degradation is dependent on any critical components of the ERAD pathway.

Interaction with K7 was found to reduce vGPCR protein, thereby dampening vGPCR-mediated signaling. Both vGPCR and K7 are expressed during KSHV lytic replication and it appears that K7 and vGPCR share an identical or overlapped expression profile. The observation that the K7 transcript peaks at a later time point than the vGPCR transcript raises the possibility that K7 serves as a negative regulatory factor to shut off vGPCR protein during KSHV lytic infection. Indeed, the knockdown of K7 by shRNA-mediated silencing increased vGPCR protein without altering vGPCR transcription level in BCBL-1 cells that are induced for KSHV lytic replication (Figure 5). Interestingly, K7 protein expression was substantially increased when co-expressed with vGPCR (unpublished data), revealing a negative feedback loop that culminates in dampening vGPCR protein expression. These observations are consistent with the notion that diverse regulatory mechanisms operate to achieve a temporary expression of vGPCR in KSHV infection. In addition to the K7-reduced vGPCR expression, known mechanisms also include the bicistronic translation and the vMIP-mediated regulation [18],[40]. Interestingly, modulation by its cognate chemokines is important for vGPCR tumorigenicity in transgenic mice [18]. Our findings that K7 interacts with vGPCR and directs it for proteasome-mediated degradation further support the notion that KSHV has evolved intricate mechanisms to regulate vGPCR activity. Additionally, K7 expression provides antiapoptotic activity under various conditions [25]–[27] and vGPCR co-expression potentiates K7's antiapoptotic activity (Figure 8F). This implies that K7 can cooperate with vGPCR in the tumorigenesis of KSHV infection, analogous to the paradigm in which Bcl-2 cooperates with c-myc [41]. However, our transformation assay in vitro and tumor growth in nude mice ruled out this possibility. Together with the biscitronic translation and modulation by vMIP chemokines, vGPCR downregulation by K7 raises an intriguing speculation that KSHV has evolved these mechanisms to monitor vGPCR pathogenicity, permitting a persistent infection within its host.

K7 expression suppressed vGPCR transformation on soft agar assay and more pronouncedly reduced vGPCR tumorigenicity in nude mice. Although K7 reduced vGPCR protein expression by approximately two-fold (Figures 6A and 8A), it was found that K7 inhibited vGPCR tumorigenicity by more than 8-fold (Figure 9B). This suggests that additional mechanisms, other than reduced protein expression, may contribute to K7's effect on vGPCR tumorigenicity. One likely mechanism is a K7-dependent retention of vGPCR in the ER, given that vGPCR predominantly localizes to the TGN and cell surface under normal circumstances. Conceivably, vGPCR functions in the TGN and on the cell surface are abolished by K7 expression. Interestingly, we have found that vGPCR is tyrosine sulfated in the TGN and tyrosine sulfation is important for vGPCR tumorigenicity (unpublished data). In addition to tyrosine sulfation, post-translational modifications in the ER (such as ubiquitination and glycosylation) altered by K7 may cause impaired vGPCR signaling and tumorigenicity. These mechanisms are not mutually exclusive and warrant further investigations of post-translational events underlying vGPCR tumorigenicity.

Mounting evidence points to vGPCR expression inducing a stress in mammalian cells including KSHV infected PEL cells [6],[13]. Indeed, our vGPCR-expressing NIH3T3 cells have a longer doubling time than control NIH3T3/vector cells (Figure 8C). Furthermore, NIH3T3/vGPCR cells gradually lost vGPCR expression when continuously passaged in vitro, suggesting that NIH3T3 cells gain a growth advantage by reducing vGPCR expression. Indeed, K7 alleviated vGPCR-mediated inhibition of NIH3T3 growth and the rate of vGPCR transcript loss (Figure 8C–8E). In contrast, vGPCR expression was necessary for tumorigenicity in nude mice, and K7-reduced vGPCR expression correlated with less transformation in vitro and tumorigenicity in vivo (Figure 9A and 9B). Interestingly, the endothelial progenitor cell line containing Bac36 (a KSHV Bacmid) behaves similarly to NIH3T3/vGPCR cells, demonstrating reduced cell growth in vitro and increased tumor formation in vivo [42]. The seemingly paradox between in vitro stress and in vivo tumorigenicity may be explained by a paracrine mechanism supported by accumulating studies [9],[43],[44]. In fact, vGPCR-induced tumor formation is highly dependent on growth factors and chemokines that stimulate the angio-proliferation of neighboring cells [43],[44]. In KS lesions, vGPCR expressing cells presumably stimulate the proliferation of spindle cells that are latently infected by KSHV. The fact that slower growth of NIH3T3 stable cell lines in vitro correlates with higher tumorigenicity in vivo suggests that the nude mice model primarily assesses the paracrine function of vGPCR. This is also supported by our in vitro transformation assay where the proliferation of regular NIH3T3 cells was examined in the presence of NIH3T3/vGPCR cells (Figure 9A). Additionally, it is not unprecedented that oncogenic proteins exploit cellular stress responses to induce tumor formation. Perhaps, these stress responses represent various barriers that oncogenesis has to overcome. For example, H-RAS triggers the ER-associated unfolded protein response, cellular senescence and sensitizes cells to apoptosis [45],[46]. Similarly, the myc-mediated stress is overcome by Bcl-2 expression [41]. Taken together, the fact that the stress in tissue culture accompanies the tumorigenicity in vivo for many oncogenic proteins suggests that the stress response may serve as an indicator for tumorigenicity in vivo. Similar to vGPCR, K7 also reduces NIH3T3 growth and it will be interesting to examine K7's tumorigenicity in nude mice.

All members of the beta- and gamma-herpesvirus family encode up to four GPCRs in their genomes. Some of them have been shown to constitutively activate signaling events downstream of various G proteins (for review see [47]). Although it was demonstrated that KSHV vGPCR can be uncoupled from downstream signal activation by overexpressed G protein-coupled receptor kinase 5 and arrestins [48], it is largely unknown how these unconventional viral GPCRs are differentially regulated as opposed to cellular GPCRs under normal physiological conditions. This study established an example of post-translational regulation of vGPCR pathogenicity by which a viral factor-induced degradation greatly influences its tumorigenicity. Similar regulatory mechanisms may exist for other viral GPCRs of herpesviruses. Therefore, viral factors that modulate these viral GPCRs likely have a profound effect on various biological activities during herpesvirus infection.

## Materials and Methods

### Plasmids

Unless specified, all constructs were derived from pcDNA5/FRT/TO (Invitrogen). A DNA fragment corresponding to the KSHV vGPCR was amplified from BCBL-1 genomic DNA by polymerase chain reaction (PCR) and cloned into pcDNA5/FRT/TO between BamHI and XhoI. For protein expression, either the HA epitope or the Flag epitope was inserted upstream or downstream of vGPCR coding sequence, respectively. Plasmids expressing wild-type and mutant K7 polypeptides were described in previous publications [25],[26]. For lentiviral expression, K7-Flag was cloned into pCDH-EF-puro (System Bioscience) between EcoRI and BamHI. HA-vGPCR was digested with EcoRI and BglII, and ligated to pCDH-EF-puro or pCDH-EF-CopGFP that was digested with EcoRI and BamHI. To generate the K7TM_StpC_, the K7 transmembrane (TM) domain was replaced with a heterologous TM segment of Stp C by PCR-based mutagenesis using overlapping PCR primers. All constructs were sequenced for verification.

For the shRNA-mediated knockdown of K7, four pairs of synthetic DNA oligos were annealed and cloned into pLKO.1 (Sigma) that was digested with AgeI and EcoRI. The pLKO.1 expressing the scrambled shRNA was purchased from Sigma. Plasmids expressing HA-tagged wt and mutant ubiquitin were a kindly gift from Dr. James Z.J. Chen (UT Southwestern).

### Cell Culture and Transfection

HEK293T (293T), HeLa, and NIH3T3 cells were grown in Dulbecco's modified Eagle's medium supplemented with 10% fetal calf serum, 5 mM L-glutamine, 100 U/ml penicillin, and 100 µg/ml streptomycin. BJAB, JSC-1, BCBL-1, and BCBL-1/T-Rex_Rta cells were grown in RPMI 1640 supplemented with 10% fetal calf serum, 5 mM L-glutamine, 100 U/ml penicillin, and 100 µg/ml streptomycin. BCBL-1 cells were treated with phorbol-12-teradecanoate-13-acetate (TPA, 20 ng/ml) to induce lytic replication. HeLa cells were transfected with Fugene 6 (Roche), 293T cells were transfected with calcium phosphate (Clontech), ECV cells were transfected with lipofectamine (Invitrogen), and BJAB cells were transfected with electroporation at 220 V/975 µF. The stable BCBL-1/T-Rex_Rta inducible cell line was maintained and induced as previously described [30].

### Immunoprecipitation and Immunoblot

Immunoprecipitation and immuno-blot analyses were performed as previously described [26]. Immunoblot detection was performed with anti-V5 antibody (1∶5000, Invitrogen), anti-Flag M2 antibody (1∶5000, Sigma), anti-HA (1∶2000, Covance), anti-tubulin (1∶250, Santa Cruz), or anti-actin (1∶30,000, Abcam). Proteins were visualized with chemical luminescent detection reagent (Pierce) and a Fuji LAS-3000 camera.

### Reverse Transcriptase (RT)-PCR

One million KSHV latently infected BCBL-1 or JSC-1 cells were treated with either TPA (20 ng/ml) to induce viral lytic replication and harvested at various time points. Alternatively, KSHV lytic replication was induced in BCBL-1/T-Rex_Rta stable cells with doxycline (1 µg/ml). Total RNA was extracted with RNAeasy column (Qiagen, CA) and digested with DNase I at 37°C for 1 h. After phenol/chloroform extraction, 1 µg of total RNA was used for first-strand cDNA synthesis using an oligo(dT) primer. Then, 1 µl of cDNA was added to 19 µl of PCR mixture and gene of interest was amplified using specific primers. PCR products were resolved on agarose gel and photographed. For each gene of interest, dilution of original cDNA and cycle number were determined to warrant that PCR products were generated within the linear range of PCR reaction. Total RNA from tumor tissues was extracted with triazol (Invitrogen, CA) and ethanol precipitation as previously described.

### Protein Stability

Transiently transfected ECV cells were pulse labeled with ^35^S-methionine/cysteine (Met/Cys) for 30 min. After extensive washing with phosphate buffered saline (137 mM NaCl, 2.7 mM KCl, 10 mM Na_2_HPO_4_, 2 mM KH_2_PO_4_, pH 7.4), cells were chased with cold medium up to 16 h. At various time points, cells were harvested, washed with cold PBS, resuspended in RIPA buffer (50 mM Tris-HCl [pH 7.4], 150 mM NaCl, 0.5% sodium deoxycholate, 0.1% SDS, 1% NP40, 5 mM EDTA/EGTA), and lysed by passing through 26-G syringe for 15 times. Centrifuged supernatant was pre-cleared with protein A/G agarose and mixed with 2 µg of anti-Flag M2 antibody. Incubation was carried out at 4°C for 4–6 h. Protein A/G agarose was added and incubation was further extended for 90 min. After extensive washing with RIPA buffer, precipitated proteins were resolved by SDS-PAGE and analyzed by autoradiography. The relative intensity of a selected protein band was quantified and its half-life was calculated. When vGPCR degradation route was investigated, 20 µM of lactacystin and MG132 (proteasome inhibitors) or 50 µM of chloriquine (a lysosome inhibitor) was added during the chase period. IP and autoradiography were performed similarly.

### Luciferase Reporter Assay

The reporter cocktail consists of plasmids expressing fire fly luciferase (50 ng/µl) and β-galactosidase (100 ng/µl). While β-galactosidase expression is driven by a housekeeping glucophosphokinase promoter, the expression of fire fly luciferase is under control of response elements of NF-κB, NF-AT, and AP-1 transcription factor. 293T cells were transiently transfected with 2.5 µl of reporter cocktail, and 200 ng of plasmids expressing vGPCR and K7. For each transfection, the total amount of plasmid was balanced with an empty vector (pcDNA5/FRT/TO). At 36 h after transfection, cells were harvested and lysed on ice. Centrifuged supernatant was used to measure luciferase and β-galactosidase activity according to manufacturer's protocol (Promega).

### Apoptosis Assay

NIH3T3 stable cells were treated with vehicle (DMSO), cyclohexamide (CHX, 1 µg/ml), or TNF-α (5 ng/ml) plus CHX (1 µg/ml) for 24 hours. Cells were harvested and live cells were scored by trypan blue staining as previously described [25]. Viable cells treated with drugs divided by viable cells treated by DMSO was used to obtain cell viability in percentage.

### Immunofluorescence Microscopy

BJAB, HeLa, or BCBL-1 cells were fixed with paraformaldehyde and permeabilized with Triton X-100 (0.2% in PBS). After stained with primary and secondary antibodies, cells were analyzed by immunofluorescence microscopy as previously described [26],[49]. vGPCR in BCBL-1 cells was detected with a gift rabbit polyclonal antibody provided by Dr. Gary Hayward [7]. For commercial antibodies, mouse monoclonal anti-Flag antibody (1∶1500), rabbit polyclonal anti-Flag antibody (1∶400, Sigma), mouse monoclonal anti-V5 antibody (1∶500, Invitrogen), sheep anti-TGN46 (1∶200, Serotec), rabbit anti-PDI (1∶200, Calbiochem) were used. All conjugated secondary antibodies were obtained from Molecular Probes and diluted at 1∶1000 (Alexa 488-conjugated) or 1∶500 (Alexa 568 or Alexa 647-conjugated).

### Knockdown of K7 by shRNA-mediated silencing

Four shRNA seuquences were designed using Dharmacon software and cloned into pLKO.1. These sequences are: 5′ TCATCCGTATTGTGTATAT 3′; 5′ CATCGTGAGTTGGTTAATA 3′; 5′ TGGCTACTCTGCTCGATTA 3′; 5′ TGAAGGATGATGTTAATGA 3′. Together with packaging plasmids DR8.9 and VSV-G, pLKO.1 plasmids expressing various K7 shRNA molecules were transfected into 293T cells with Fugene 6 (Roche). Lentivirus expressing the scrambled shRNA was produced similarly. Filtered lentivirus was used to infect BCBL-1 cells at 20 MOI in medium containing 10 µg/ml polybrene. To increase infection efficiency, cells were centrifuged at 1,800 rpm, 30°C for 1 h and incubation was further extended for up to 12 h. The infection was repeated once and cells were selected with puromycin at 1 µg/ml. At 48 h later, BCBL-1 cells were treated with TPA (20 ng/ml) to induce KSHV lytic replication.

### Cell Growth and Soft Agar Assay

NIH3T3 cells were infected with lentiviruses to establish stable cell lines expressing K7 with puromycin selection. Then, NIH3T3/puro and NIH3T3/K7 cells were further infected with lentivirus expressing GFP or vGPCR. This lentiviral infection was repeated once to obtain stable cells expressing K7, vGPCR, or vGPCR and K7. Cells were cultured in complete DMEM medium containing puromycin (1 µg/ml). To measure the doubling time, 2×10^5^ cells were plated and cells were counted at 24 h and 48 h later. The soft agar assay was performed as described by Liang et al [50]. Stable NIH3T3 cells (5×10^4^) were mixed with 1×10^5^ normal NIH3T3 cells and cultured for two weeks in regular culture medium without puromycin.

### Tumor Formation In Vivo

All animal experiments were performed according to the National Institutes of Health principles of laboratory animal care and approved by the University of Texas Southwestern Medical Center. Stable NIH3T3 cells (3×10^6^/site) expressing GFP, K7, vGPCR, or vGPCR and K7 were injected subcutaneously into the flanks of 6- to 8-wk-old mice (athymic, *nu*/*nu*, Jackson Laboratory).

## Supporting Information

Figure S1 Knockdown of K7 During KSHV Lytic Reactivation. (A) The knockdown efficiency of K7 by shRNA-mediated silencing. BCBL-1 cells were infected with lentivirus and induced for lytic reactivation as diagrammed in Figure 4D. RT-PCR analyses were performed with serial dilution of cDNA template (shown in Figure 4D) as indicated by the ratio. (B) K7 knockdown on cell viability in KSHV lytic reactivated cells. Lentivirus infection and lytic reactivation were performed as in (A). Cells were harvested and cell viability was assessed by trypan blue staining.
(0.12 MB TIF)

Figure S2 Reduced Expression of vGPCR by K7 Mutants and Lactacystin Treatment. (A) Glycosylation and ubiquitination are dispensable for K7’s ability to reduce vGPCR protein expression. Whole cell lysates of ECV cells transfected with plasmids containing vGPCR and K7 as indicated were analyzed by immunoblot with anti-Flag (vGPCR, top panel), anti-actin (middle panel), and anti-V5 (K7, bottom panel). Of note, the K7(5K>R) and K7(N108Q) carry 6xHIS downstream of the V5 epitope that reduces their detection by immunoblot. Ub, ubiquitinated K7; gly, glycosylated K7. (B) The effect of lactacystin treatment on K7-reduced vGPCR expression. Human ECV cells were transfected with plasmids containing vGPCR or K7 and treated for 6 h with DMSO or lactacystin (20 μM). Whole cell lysates were analyzed by immunoblot with anti-Flag (vGPCR, top panel), anti-actin (middle panel), and anti-V5 (bottom panel).
(0.17 MB TIF)

Figure S3 Relative Expression Levels of vGPCR in Reactivated BCBL-1 Cells and NIH3T3 Stable Cells. Total RNA was extracted and RT-PCR analyses were performed as described in Materials and Methods using gene specific primers to vGPCR and β-actin. BCBL-1 cells were induced for lytic reactivation by TPA (20 ng/ml, 48 h) or BCBL-1/T-Rex_Rta cells were treated with doxycycline (1 μg/ml, 72 h) before harvest. No PCR product was etected for controls without RT (data not shown). The primers for β-actin locate within a highly conserved region of the human and mouse β-actin gene.
(0.11 MB TIF)

Figure S4 vGPCR mRNA Levels in NIH3T3 Stable Cells by Real-Time PCR. The primers were designed using Primer Express v1.5 (Applied Biosystems). The efficiency and specificity of primers were validated and the real-time PCR using cDNA was performed with an ABI 7500 sequence detection system (Applied Biosystems). The vGPCR mRNA level at day 7 was arbitrarily set as 1. Data represent three independent experiments and error bars denote standard deviation.
(0.05 MB TIF)
