# Supplementary figures and images for: Kaposi's Sarcoma-Associated Herpesvirus K7 Induces Viral G Protein-Coupled Receptor Degradation and Reduces Its Tumorigenicity
Source: PLoS Pathog. 2008 Sep 19;4(9):e1000157. doi: 10.1371/journal.ppat.1000157 (PMC2529400; doi:10.1371/journal.ppat.1000157)

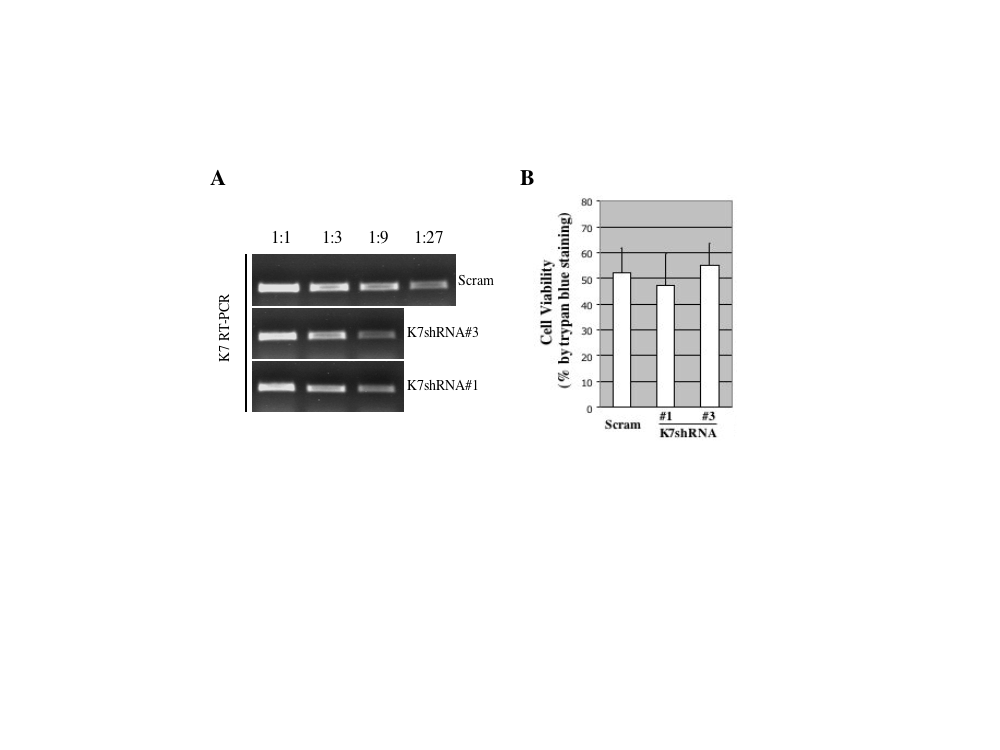

Supplement: Figure S1 — Knockdown of K7 During KSHV Lytic Reactivation. (A) The knockdown efficiency of K7 by shRNA-mediated silencing. BCBL-1 cells were infected with lentivirus and induced for lytic reactivation as diagrammed in Figure 4D. RT-PCR analyses were performed with serial dilution of cDNA template (shown in Figure 4D) as indicated by the ratio. (B) K7 knockdown on cell viability in KSHV lytic reactivated cells. Lentivirus infection and lytic reactivation were performed as in (A). Cells were harvested and cell viability was assessed by trypan blue staining. (0.12 MB TIF) [file ppat.1000157.s001.tif]

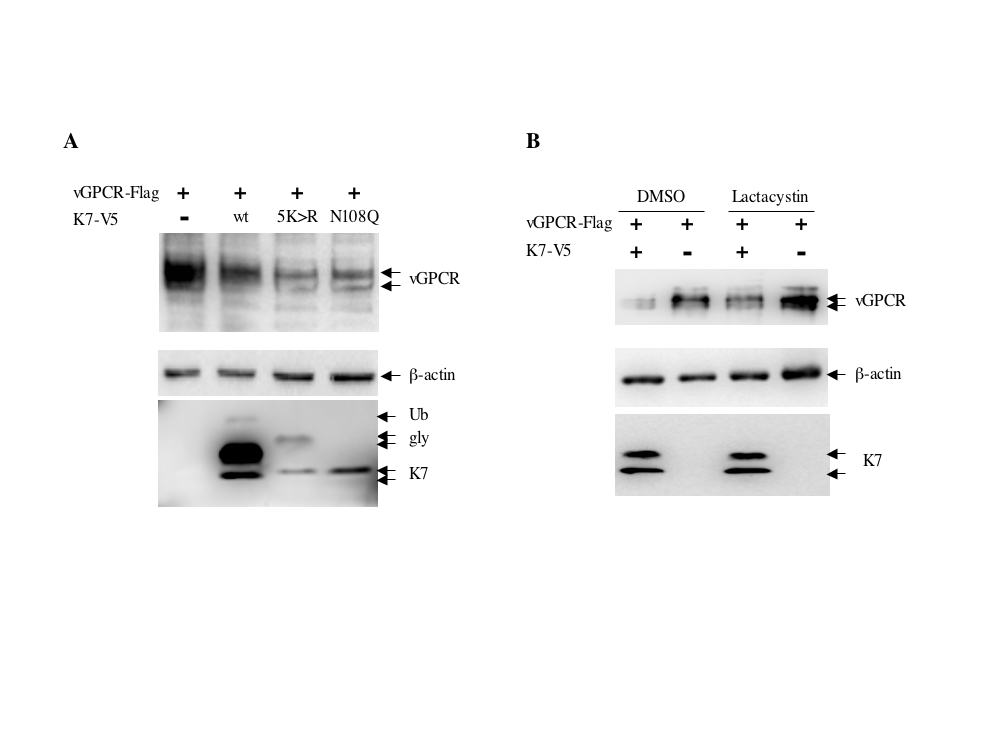

Supplement: Figure S2 — Reduced Expression of vGPCR by K7 Mutants and Lactacystin Treatment. (A) Glycosylation and ubiquitination are dispensable for K7’s ability to reduce vGPCR protein expression. Whole cell lysates of ECV cells transfected with plasmids containing vGPCR and K7 as indicated were analyzed by immunoblot with anti-Flag (vGPCR, top panel), anti-actin (middle panel), and anti-V5 (K7, bottom panel). Of note, the K7(5K>R) and K7(N108Q) carry 6xHIS downstream of the V5 epitope that reduces their detection by immunoblot. Ub, ubiquitinated K7; gly, glycosylated K7. (B) The effect of lactacystin treatment on K7-reduced vGPCR expression. Human ECV cells were transfected with plasmids containing vGPCR or K7 and treated for 6 h with DMSO or lactacystin (20 μM). Whole cell lysates were analyzed by immunoblot with anti-Flag (vGPCR, top panel), anti-actin (middle panel), and anti-V5 (bottom panel). (0.17 MB TIF) [file ppat.1000157.s002.tif]

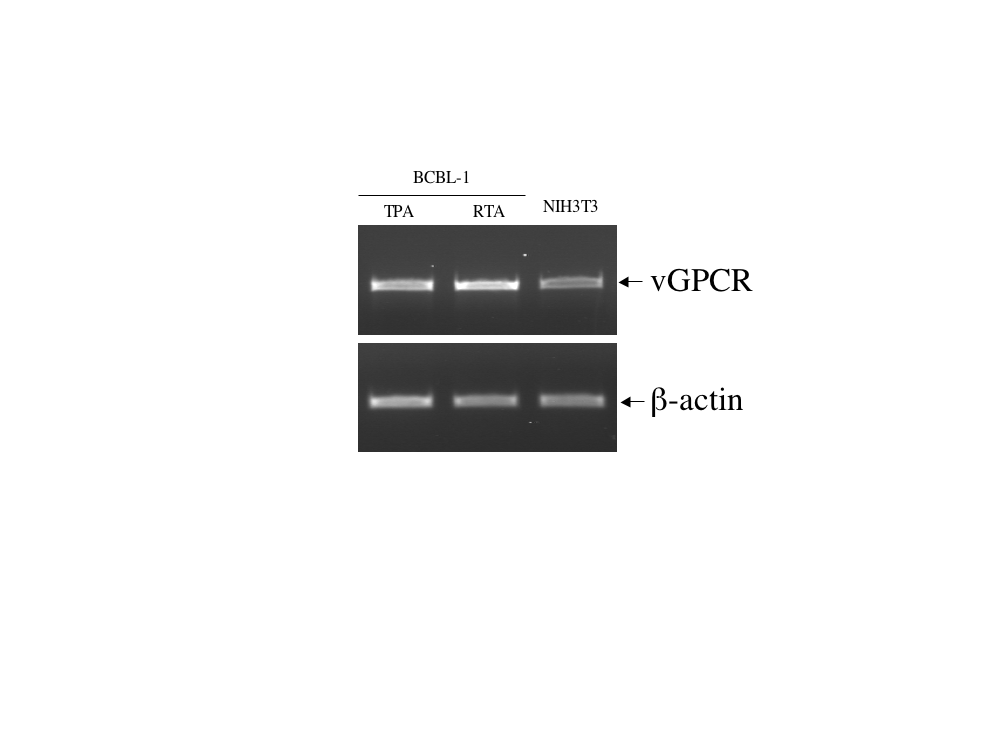

Supplement: Figure S3 — Relative Expression Levels of vGPCR in Reactivated BCBL-1 Cells and NIH3T3 Stable Cells. Total RNA was extracted and RT-PCR analyses were performed as described in Materials and Methods using gene specific primers to vGPCR and β-actin. BCBL-1 cells were induced for lytic reactivation by TPA (20 ng/ml, 48 h) or BCBL-1/T-Rex_Rta cells were treated with doxycycline (1 μg/ml, 72 h) before harvest. No PCR product was etected for controls without RT (data not shown). The primers for β-actin locate within a highly conserved region of the human and mouse β-actin gene. (0.11 MB TIF) [file ppat.1000157.s003.tif]

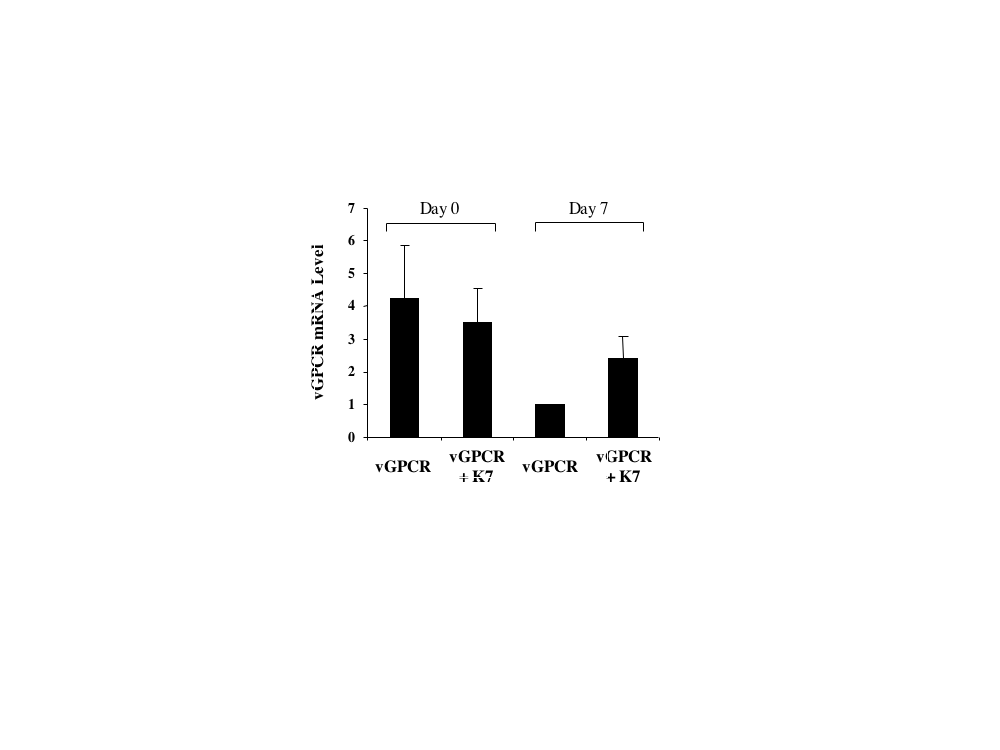

Supplement: Figure S4 — vGPCR mRNA Levels in NIH3T3 Stable Cells by Real-Time PCR. The primers were designed using Primer Express v1.5 (Applied Biosystems). The efficiency and specificity of primers were validated and the real-time PCR using cDNA was performed with an ABI 7500 sequence detection system (Applied Biosystems). The vGPCR mRNA level at day 7 was arbitrarily set as 1. Data represent three independent experiments and error bars denote standard deviation. (0.05 MB TIF) [file ppat.1000157.s004.tif]
